# Examining the relationship between sexual dimorphism in skin anatomy and body size in the white-lipped treefrog, *Litoria infrafrenata* (Anura: Hylidae)

**DOI:** 10.1093/zoolinnean/zly070

**Published:** 2019-11-06

**Authors:** Collin S. Vanburen, David B. Norman, Nadia B. Fröbisch

**Affiliations:** 1 Department of Earth Sciences, University of Cambridge, Cambridge, UK; 2 Museum für Naturkunde, Berlin, Germany; 3 Institute for Biology, Humboldt University of Berlin, Berlin, Germany

**Keywords:** amphibians, ecomorphology, histology, Hylidae, sexual dimorphism

## Abstract

Amphibians transport water, oxygen, carbon dioxide and various ions (e.g. sodium and potassium) across their skin. This cutaneous permeability is thought to affect their ability to respond to environmental change and to play a role in global population declines. Sexual dimorphism of skin anatomy has been accepted in some species, but rejected in others. The species in which such dimorphism has been detected have all been sexually dimorphic in body size, with males that are smaller and have thinner skin. It is unclear whether this difference in skin thickness manifests a functional difference or if it is related to body size alone. Skin thickness (epidermis, spongy dermis, compact dermis and total thickness) was examined in males and females of the white-lipped treefrog (*Litoria infrafrenata*). Although the skin of males is absolutely thinner than that of females, this difference is explained by body size differences between the sexes. Overall, we conclude that skin thickness in male and female *L. infrafrenata* correlates with body size dimorphism and suggest that future studies on amphibian skin anatomy include measures of body size, test the ecological significance of sexually dimorphic skin anatomy and better document the prevalence of sexually dimorphic amphibian skin anatomy.

## Introduction

The skin of amphibians is semipermeable and allows gases, liquids and ions (e.g. sodium and potassium) to be exchanged between the internal tissues and external environment (Duellman & Trueb, 1986). The permeability of amphibian skin renders animals susceptible to desiccation through evaporative water loss; the skin is so ‘leaky’ that this physiological property is sometimes used to explain why a higher proportion of amphibian species are threatened with extinction compared to other terrestrial vertebrate clades that are better able to regulate their control flux through their skin (Wake & Vredenburg, 2008). Interspecific (Le Quang Trong, 1971, 1975; Ponssa *et al*., 2017) and intraspecific (Kun, 1959; Kobelt & Linsenmair, 1986; Wenying *et al*., 2011) studies on amphibian skin anatomy suggest that variation in its structure is related to ecology or physiology. For example, the skin of the reed frog (*Hyperolius nitidulus*) is thicker in the dry season than it is in the wet season, which helps it to reduce evaporative water loss (Geise & Linsenmair, 1986; Kobelt & Linsenmair, 1986) and populations of the Cururu toad (*Rhinella schneideri*) from different habitat types differ in skin thickness and texture (Navas *et al*., 2004). However, relatively little is known about anatomical variation in skin characteristics across the entire clade or their direct functional significance.

Many sources of intraspecific variation in skin anatomy exist, including seasonal variation and sexual dimorphism. Previous research on sexually dimorphic skin microanatomy has focused on specialized glands unique to males (e.g. Sever 1976, 1989; *Brunetti et al*. 2015). These glands, when present, are found in the mental (chin) region, as well as the tail of salamanders (Weichert, 1945; Sever, 1976, 1989) and the lateral region of frogs (Brunetti *et al*., 2015). Their function is unknown but is likely related to mating because they become enlarged during the breeding season (Weichert, 1945; Sever, 1976, 1989).

Skin thickness is a trait thought to affect skin physiological function (Toledo & Jared, 1993) and is also known to vary between the sexes. Male African clawed frogs (*Xenopus laevis*) and some species in the genus *Ptychadena* have thinner skin than females (Le Quang Trong, 1975; Greven *et al*., 1995); conversely, male Siberian wood frogs (*Rana amurensis*) have thicker skin in the breeding season than females (Wenying *et al*., 2011). In Dybowsky’s frog (*Rana dybowskyii*), there is no consistent pattern across the body, where females have thicker dorsal skin and males have thicker ventral and lateral skin (*Lili et al*., 2013), and in the cane toad (*Rhinella marinus*) and the green frog (*Pelophylax esculentus*), the sexes do not differ in skin thickness (Zanger *et al*., 1995; Schwinger *et al*., 2001). Absolute and relative differences in skin thickness between males and females may be ecologically significant because skin morphology traits are linked to the ability of amphibians to transfer substances through the skin (McClanahan & Baldwin, 1969; Roth, 1973; Boutilier *et al*., 1986; Katz, 1986; Toledo & Jared, 1993). Therefore, if these anatomical traits differ between males and females, then the two sexes might differ in physiology, microhabitat preferences or fundamental niche.

Although amphibian skin anatomy has been studied for over 150 years (Ascherson, 1840), integrative studies seeking to answer broad evolutionary and ecological questions about this structure are lacking. Sexual dimorphism in body size is pervasive among amphibians (e.g. De Lisle & Rowe, 2015), yet previous studies of skin anatomy have not corrected for these sometimes extreme differences in body size. Larger frogs take longer to dehydrate to dangerous levels than smaller frogs but also take longer to rehydrate (*Tracy et al*., 2010), a process that is thought to be mediated in part by skin thickness (Toledo & Jared, 1993). Among species of the African grassland frogs (genus *Ptychadena*), savannah species seem to have relatively thicker skin and smaller body size than species inhabiting forest or mixed habitats (Le Quang Trong, 1975). Conversely, among puddle frogs (genus *Phrynobatrachus*), body size and skin thickness seem to co-vary with habitat type (Le Quang Trong, 1971). The taxonomic breadth of each of these studies is small, so drawing broad conclusions should be done with caution. Taken together, however, these data suggest that skin thickness has ecological significance and reinforces the need for more rigorous studies on inter- and intraspecific variation in skin anatomy in order to clarify these relationships and interrelationships.

To investigate sexual dimorphism in the skin thickness of amphibians, we examined the skin of the whitelipped treefrog (*Litoria infrafrenata*), which is native to the wet tropical forests of South-East Asia and Australia. This species was chosen because it exhibits body size sexual dimorphism and is a close relative of the Australian green treefrog (*L. caerulea*), which is used commonly in laboratory-based studies of amphibians (e.g. Buttemer 1990; Christian & Parry, 1997; Voyles *et al*., 2009). Moreover, the current study represents the first on skin anatomy sexual dimorphism of a terrestrial tropical rainforest amphibian.

## Material and Methods

### Specimens and preparation

We sampled eight formalin-fixed, alcohol-preserved specimens of *Litoria infrafrenata* from the collections of the Museum für Naturkunde (MfN) in Berlin, Germany. All specimens were collected near Seru on the island of Yapen, Indonesia, on 27 August 1995. Because the specimens were all collected on the same day and appear to represent full-grown adults, we are able to discount seasonal or ontogenetic effects, which are known to affect skin anatomy (Kun, 1959; Kobelt & Linsenmair, 1986; Rosenberg & Warburg, 1995). Body size was measured using snout-vent length (SVL). Three of the specimens were male (SVL 67-72 mm) and five were female (SVL 90-105 mm). Specimens were sexed and aged upon collection by R. Günther.

**Figure 1:**
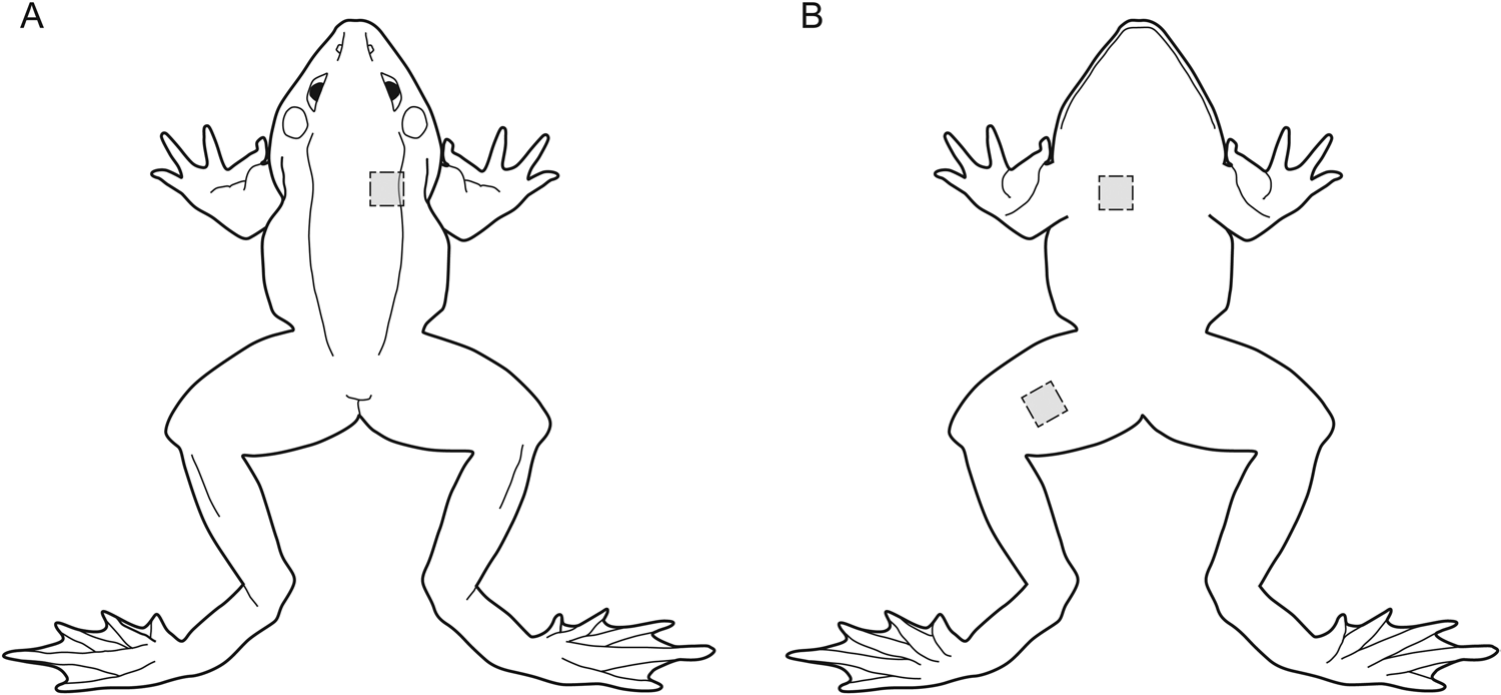
Sampling locations across the body. The locations where the dorsal (A, dorsal pectoral) and ventral (B, ventral pectoral and ventral thigh) skin was sampled. Sampling regions are indicated by the grey boxes.

Skin biopsies roughly 0.5 cm^2^ in size were taken from three regions: the dorsal pectoral, ventral pectoral and ventral thigh regions on the right-hand side of the body. The dorsal pectoral and ventral pectoral regions were chosen because they are commonly sampled in other studies on amphibian skin anatomy (e.g. Greven *et al*. 1995; Zanger *et al.* 1995) and the ventral thigh region was selected because of the function of this area of skin for water absorption in at least some anurans (Roth, 1973). Dorsal pectoral and ventral pectoral samples were taken close to the pectoral girdle and adjacent to the midline; ventral thigh samples were taken from the ventral surface near the midshaft of the femur (Fig. 1).

The methodology used to prepare the specimens for museum storage is unknown. In an attempt to create a more ‘life-like’ skin thickness, and to reduce the effect of alcohol-induced shrinkage, we first rehydrated the skin samples by allowing them to sit in decreasing concentrations of alcohol (70%, 50%, 30%) and finally phosphate-buffered saline solution (PBS) for an hour each before being placed in 4% formalin overnight. We then placed the specimens in PBS for an hour before being progressively dehydrated and embedded in paraffin wax, which is a standard protocol for preparing fresh tissues for histological preparation (Bancroft & Gamble, 2008). Although chemically mediated preservation protocols and histological preparation may be expected to shrink soft tissue, all eight specimens were stored in the same jar in the collections and were prepared using the same methodology. We expect that any preservation or preparation biases will affect all specimens in a similar way, and therefore reduce their effects on the overall results. Sections were made at 5-pm thickness using a Leica SM2000 R Sliding microtome and then stained using Azan staining modified after Geides (Geidies, 1954) and Masson Goldner’s Trichrome (Goldner, 1938).

### Data collection

**Figure 2:**
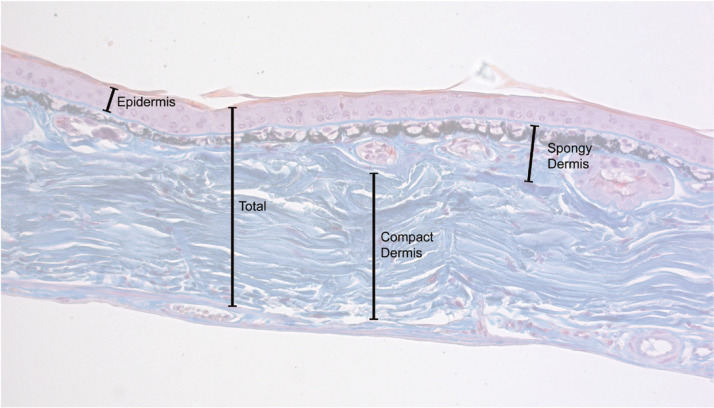
Histology measurements. Examples of how the various thickness measures were taking on images of stained tissue.

Photographs of the histological sections were taken with a Leica DFC490 camera mounted on an Axioskop light microscope and then measured in the program ImageJ (Abramoff *et al*., 2004). Linear measurements were recorded of the thickness of the epidermis, spongy dermis and compact dermis, as well as capillary depth. To capture spatial heterogeneity in tissue thickness, ten measurements were taken randomly across the series of images for each specimen. The thickness of the epidermis was measured orthogonally from the basement membrane (stratum basale; Fig. 2). The thickness of the spongy and compact dermis was measured using a line orthogonal to the orientation of the connective tissue layers in the compact dermis (Fig. 2). These methods for measuring skin thickness are similar to those used by Ponssa *et al.* (2017). Epidermis thickness was also measured by counting the number of cells between the basement membrane and the surface of the skin.

### Analysis

To test for a relationship between skin thickness and body size, we first calculated Spearman’s *rho* between each skin thickness measurement and SVL for all specimens. We also tested for this relationship within each sex. Because the dataset is small and does not meet assumptions of parametric tests (e.g. normality), we used nonparametric methods to test for differences between males and females. We first used a Kruskal- Wallis test to test for differences in uncorrected skin thickness measures between the sexes using the mean values for each measurement. We then calculated residual values from a regression of means of each skinthickness measure against body size to remove effects of size from our data and performed a second set of Kruskal-Wallis tests. Unfortunately, there was no linear relationship between body size and skin thickness for any variable in males (likely due to the low sample size) and only for a few variables in females (Supporting Information, Table S1, Fig. S1). Thus, methods such as linear mixed effects models that utilize multiple measurements per specimen (with specimen ID included as a random effect), but assume a linear relationship within groups, were inappropriate for our data. All analyses were performed using R (R Development Core Team, 2016).

## Results

### Description of Sampled Skin Regions and Results from Statistical Tests of Skin Thickness

**Figure 3:**
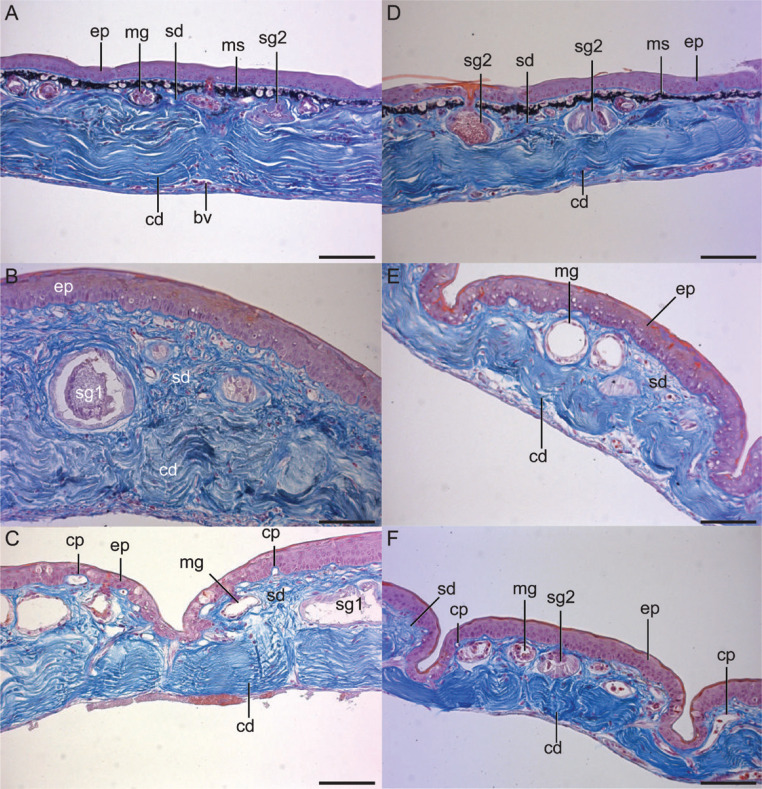
Histological sections of the skin of male and female *Litoria infrafrenata.* The skin of female (A-C) and male (D-F) *L. infrafrenata* is shown, sampled from the dorsal (A, MfN 54644; D, MfN 54644), ventral (B, MfN 54637; E, MfN 54642), and thigh (C, MfN 54647; F, MfN 54642) regions of the body; cd = compact dermis, cp = capillary, ep = epidermis, mg = mucous gland, ms = melanosomes, sd = spongy dermis, sg1 = serous gland type 1, sg2 = serous gland type 2. Scale bar = 100 pm.

In both sexes, the skin is composed of the three standard cutis tissue layers: the epidermis, spongy dermis and compact dermis (Fig. 3). When the size-uncorrected values were analysed, males display significantly thinner compact dermis in the dorsal pectoral region; thinner epidermis, spongy dermis and compact dermis in the ventral pectoral region; and thinner spongy dermis and compact dermis in the ventral thigh region (Table 1). The total skin thickness in all three of the sampled body regions is less in males than in females before correcting for body size (Table 1). Most of these thickness measures were also significantly correlated with body size (Table 2). Only dorsal pectoral epidermis and spongy dermis thickness and ventral pectoral epidermis thickness did not have significant relationships (Table 2). When size-corrected values of skin thickness were compared between males and females, no significant differences were found (Table 1) and no relationship was found between skin thickness and body size (Table 2).

**Table 1:** Average skin thickness of male and female *Litoria infrafrenata* compared using Kruskal-Wallis tests both on the raw data and residuals from regressions of skin thickness vs. SVL. Skin thickness reported in μm

Region	Layer	Average (male)	Average (female)	Raw data			Residuals		
				Chi-squared	df	*P*-value	Chi-squared	df	*P*-value
Dorsal	All	164	248.8	5	1	0.03	0.022	1	0.88
	Epidermis	24.9	25.9	0.022	1	0.88	0.022	1	0.88
	Spongy Dermis	50.6	62.6	0.2	1	0.65	0.022	1	0.88
	Compact Dermis	88.5	160.2	5	1	0.03	0.022	1	0.88
Ventral	All	188	309.9	5	1	0.03	0.2	1	0.65
	Epidermis	40.5	53.5	5	1	0.03	0.022	1	0.88
	Spongy Dermis	64	103.1	5	1	0.03	0.022	1	0.88
	Compact Dermis	83.5	153.3	5	1	0.03	0.56	1	0.46
Thigh	All	167.4	295.3	5	1	0.03	0.022	1	0.88
	Epidermis	32	39.8	3.76	1	0.052	0.56	1	0.46
	Spongy Dermis	59.2	119.6	5	1	0.03	1.8	1	0.18
	Compact Dermis	76.3	135.9	5	1	0.03	0.022	1	0.88

**Table 2:** Results from Spearman’s correlation tests comparing skin thickness measures to SVL

		All	*P*-value	Males	*P*-value	Females	*P*-value
		rho		rho		rho	
Dorsal	All	0.81	0.02	0.5	1	0.3	0.68
	Epidermis	-0.12	0.79	0.5	1	-0.9	0.08
	Spongy Dermis	0.33	0.43	0.5	1	0.3	0.68
	Compact Dermis	0.83	0.02	0.5	1	0.3	0.68
Ventral	All	0.88	0.007	0.5	1	0.9	0.08
	Epidermis	-0.57	0.15	0.5	1	0.3	0.68
	Spongy Dermis	0.79	0.03	0.5	1	0.8	0.13
	Compact Dermis	0.81	0.02	0.5	1	0.6	0.35
Thigh	All	0.95	0.001	0.5	1	0.6	0.35
	Epidermis	0.81	0.02	0.5	1	-0.2	0.78
	Spongy Dermis	0.93	0.002	0.5	1	0.1	0.95
	Compact Dermis	0.88	0.007	0.5	1	0.3	0.68

#### Dorsal skin sample: pectoral region

Based on average skin-layer thicknesses (Table 1), the dorsal pectoral skin is the thinnest of the three regions sampled. However, it should be noted that the total thicknesses of the three regions do not significantly differ from one another (Supporting Information, Table S2). The thickness of the tissue layers is uniform across the sampled region. The epidermis is 3-5 cells thick in females and 3-6 cells thick in males. The dorsal pectoral region is the only region that was sampled that had clearly defined melanosomes and melanocytes (Fig. 3A, D). The melanocytes are superficial to the melanin-filled melanosomes that they produce.

#### Ventral skin sample: pectoral region

Unlike the skin of the dorsal pectoral region, the ventral region is marked by vercuae: regions of expanded spongy and compact dermis separated by troughs of thin dermis and slightly thinner epidermis (Fig. 3B, E). The vercuae are thicker and wider in females than they are in males. Skin in the ventral region is the thickest on average; however, it becomes much thinner in all three tissue layers in the troughs between the vercuae (Fig. 3B, E). The epidermis is 3-7 cells thick in males and 3-8 cells thick in females, but it is 3-5 cells thick in the troughs between vercuae in both sexes and 5-7 cells thick at the apex of the vercuae in males and 5-8 cells thick at the apex in females. Blood vessels within the spongy dermis lie directly against the basement membrane, extending the blood vessel superficially into the region normally occupied by the epidermis at various points in the vercuae but never break through the basement membrane. The number of cells in the epidermis superficial to the blood vessels in these regions is lower than when blood vessels are not present.

#### Ventral thigh skin sample

Vercuae are present, much like those seen in the ventral pectoral region, and they are again smaller in males than they are in females (Fig. 3C, F). The epidermis is 3-6 cells thick in males and 3-7 cells thick in females. Like in the ventral pectoral skin, the epidermis contains fewer cells in regions between vercuae, as well as above where blood vessels sit directly below the basement membrane of the epidermis.

### Description of Gland Types Across Sampled Skin Regions

Three types of glands can be distinguished in the sampled skin regions. Mucous and serous (granular) glands are conspicuous, and serous glands can be subdivided into two distinct types described by Delfino *et al.* (1998) (Fig. 3). Mucous glands are typical of other amphibians and are characterized by possessing a thin epithelium and relatively small lumen, compared with the serous glands; this difference is more pronounced in females than in males (Fig. 3C, F). The nuclei and cytoplasm of the mucous gland epithelial cells are more reactive to Azan staining, appearing darker in colour than the epithelial cells of either of the serous gland morphotypes (Fig. 3A, C, E, F). The cells of mucous glands are also smaller and more ovoid compared to the elongate epithelial cells of serous glands. The first type of serous gland (Type 1a, Delfino *et al.* 1998) is similar to that of all other amphibians whose gland morphology has been studied (Fig. 3B, C). Each has a thin epithelium and a relatively large lumen, usually filled with granules (Fig. 3B, C). The second type of serous gland (Type 1b or II, Delfino *et al*., 1998) has relatively thick, bulbous epithelial cells that stain a lighter shade of pink than the other two types of glands with the Azan stain; they contain non-uniform granules that are roughly twice the size of the granules in the first serous gland (Fig. 3A, D, F). The anatomy of this second type of serous gland is similar to that of polymorphic serous glands reported in other hylids (Delfino *et al*., 1998). On this basis, we propose that two serous gland types and one mucous gland type may be present in the skin of *L. infrafrenata*.

Glands are more densely distributed in the dorsal region than in the ventral or thigh regions. The first type of serous gland was observed much less frequently in samples from females, as we only detected them in a single female specimen (MfN 54646). However, they were found in samples from all male specimens. All three gland types were prevalent in both of the ventral skin samples. There are no sexually dimorphic glands identified in the skin regions we sampled.

### Capillaries

Capillaries have a very thin lumen and are usually identifiable because they still retain blood cells that stain bright red with Azan staining. They are usually present in the superficial spongy dermis, and displace the epidermis in the ventral pectoral and ventral thigh regions by migrating into the space occupied by epidermal cells but never breaking the basement membrane. Blood vessels enter the spongy dermis through collagenous columns ascending from the hypodermis (Azevedo *et al*., 2006).

In the ventral pectoral and ventral thigh regions, blood vessels enter the space normally occupied by the epidermis without separating the basement membrane. This feature was so extreme in the ventral thigh region of one male (MfN 54641) that the basement membrane was difficult to identify, obscuring the separation between the epidermis and spongy dermis. In this specimen, the more highly vascularized regions of the epidermis were much thicker than the non-vascularized regions, and may be indicative of an unknown pathology. We excluded this specimen from statistical analyses of skin thickness measures to determine its effect on the results. When MfN 54641 was removed, there was no difference in the number of significant differences between groups (Supporting Information, Table S3).

## Discussion

In this study, we describe the skin microanatomy of the white-lipped treefrog, *Litoria infrafrenata*, and test for sexual dimorphism in skin anatomy using linear morphometrics. Overall, the skin microanatomy of *Litoria infrafrenata* is similar to that of other anurans, consisting primarily of an epidermis, spongy dermis, compact dermis, and both mucous and serous glands (Fox, 1986a, b). Apart from skin thicknesss, there were no histochemical or anatomical differences between the sexes that have been described in other species, such as sexually dimorphic skin glands. One notable feature is the presence of polymorphic serous glands, which are distinguished from one another based on features such as lumen and granule morphology. Similar polymorphisms in serous or mucous glands have been described in other hylids (Delfino *et al*., 1998; Centeno *et al*., 2015). Polymorphic skin glands in anurans are of particular interest because, in species whose gland histochemistry and function have been studied, their secretions significantly reduce evaporative water loss (EWL) to rates comparable to amniotes (Shoemaker *et al*., 1972). However, methods for differentiating between these gland types are not consistent among researchers, and include both histochemical and microscopy approaches (Blaylock *et al*., 1976; Delfino *et al*., 1998, 2002, 2006; Warburg *et al*., 2000; Barbeau &Lillywhite, 2005; Centeno *et al*., 2015). A standard protocol for classifying polymorphic skin glands is lacking, but such a protocol will be required in order to better understand the evolution and function of polymorphic skin glands among amphibians.

As predicted, uncorrected measures of skin thickness in *Litoria infrafrenata* differ between males and females, with females having thicker skin (Table 1). Females also attain larger adult body size, so this relationship mimics what has been reported in adults of the African clawed frog, *Xenopus laevis* (*Greven et al*., 1995). Unlike previous research, we tested for differences in each tissue layer in the body regions examined. Surprisingly, we found no difference between males and females in the dorsal pectoral epidermis or spongy dermis, even though total skin thickness was significantly different between the sexes. The dorsal surface of the body is the most exposed to the external environment, whereas the ventral surface can be protected through behaviours like the water-conserving posture (Heatwole, 1963; Barbeau & Lillywhite, 2005). Under standard conditions, amphibians lose water through passive EWL at higher rates than other tet- rapods, and a variety of adaptations to mitigate EWL have likely evolved multiple times (Toledo & Jared, 1993). Previous work has shown that rates of EWL are inversely proportional to body size (Tracy *et al*., 2010). Therefore, the smaller male *Litoria infrafrenata* may be under selective pressure to have relatively thick dorsal epidermis and spongy dermis to help protect against evaporative water loss. These results also suggest that, among the three tissue layers, the epidermis and spongy dermis might be more related to rates of EWL than the compact dermis. It is possible that the mechanism for increasing epidermal thickness is to increase the number of cells, but this should be tested interspecifically.

We also predicted that body size would explain the differences in skin thickness between males and females. We used two approaches to test for the effects of body size on skin thickness. First, using a Spearman’s rank-order correlation test, we found that most measures of skin thickness across tissue layers and body regions were significantly correlated with SVL (Table 2). These significant relationships support the hypothesis that sexually dimorphic skin thickness is explained by body size differences. Unfortunately, the range of within-group body size variation and small sample size made it impossible to test for differences in slope or intercept values between the sexes using our dataset, particularly for the males (*N* = 3). We also used residual values from regressions of skin thickness against SVL to test for differences between the sexes and found that females were no longer significantly different from males in any measure (Table 1). Taken together, these results support the hypothesis that body size explains the difference in skin thickness between male and female *Litoria infrafrenata*.

Previous work on sexual dimorphism in amphibian skin thickness has failed to find consistent patterns across species. When examined together, most of these studies found sexual dimorphism in skin thickness only existed in species in which body size was also sexually dimorphic (Greven *et al*., 1995; *Zanger et al*., 1995; Schwinger *et al*., 2001). Our study is the first to consider this relationship quantitatively and we find that body size explains sexual dimorphism in skin thickness. Furthermore, studies that found different patterns within species may have done so due to their sampling method. For example, males of *Rana amu- rensis* sampled in the breeding season were found to have thicker skin than females in some body regions, but this difference is thought to be explained by the presence of seasonally enlarged sexually dimorphic skin glands in males (Wenying *et al*., 2011). Our dataset, however, contains individuals sampled at the same time that do not show signs of specialized breeding structures.

This study represents the first attempt to quantify the relationship between sexual dimorphism in skin anatomy and in body size for anuran amphibians. Although the sample size is small, it is relatively large for a histological study (e.g. Bingol-Ozakpinar & Murathanoglu, 2011). Our findings suggest that the differences in skin thickness between males and females in *Litoria infrafrenata* are due to differences in body size. Our results also suggest that the thickness of the epidermis and spongy dermis might be more related to rates of EWL than the compact dermis. However, this prediction needs to be tested. Future research should investigate patterns of sexual dimorphism in skin thickness in other species (e.g. *Xenopus laevis),* particularly by sampling males and females at various sizes so that methods such as linear mixed effects models can be applied to the data. Furthermore, other sources of variations, such as seasonal skin thickening, also warrant investigation to test the potential effect of these factors on the study of amphibian skin.

## Supporting Information

**Figure S1**. Regressions of skin thickness against body size. Regressions of each skin thickness measure (labelled left) in each body region (labelled top) against body size. Black points are females and grey points are males. The solid black line is the regression line for all specimens, the broken black line is for females, and the broken grey line is for males. Both axes are log-transformed.

**Table S1**. Summary statistics for regressions of skin thickness measurements vs. SVL for all specimens, females, and males. Shaded boxes represent significant values (P < 0.05).

**Table S2**. Comparing total thickness between body regions.

**Table S3**. Average skin thickness of male and female *Litoria infrafrenata* compared using Kruskal-Wallis tests both on the raw data and residuals from regressions of skin thickness vs. SVL with the pathological specimen (MfN 54646) removed.

## References

[cit0001] Abràmoff MD, Magalhães PJ, Ram SJ 2004 Image processing with ImageJ. *Biophotonics International* 11: 36–42.

[cit0002] Ascherson FM 1840 Über die Hautdrüsen der Frösche. In: *Über die Hautdrüsen der Frösche und über die Bedeutung der Fettstoffe zwei physiologische Abhandlungen*. Berlin: Verlag von Veit und Comp, 3–11.

[cit0003] Azevedo RA, de Jesus Santana AS, de Brito-Gitirana L 2006 Dermal collagen organization in Bufo ictericus and in *Rana catesbeiana* integument (Anuran, Amphibian) under the evaluation of laser confocal microscopy. *Micron* 37: 223–228. 16376554 10.1016/j.micron.2005.11.001

[cit0004] Bancroft JD, Gamble M 2008 *Theory and practice of histological techniques*. London: Churchill Livingstone/Elsevier.

[cit0005] Barbeau TR, Lillywhite HB 2005 Body wiping behaviors associated with cutaneous lipids in hylid tree frogs of Florida. *The Journal of Experimental Biology* 208: 2147–2156. 15914658 10.1242/jeb.01623

[cit0006] Bingol-Ozakpinar O, Murathanoglu O 2011 The morphology of the dorsal and ventral skin of *Triturus karelinii* (Caudata: Salamandridae). *Biologia* 66: 349–356.

[cit0007] Blaylock LA, Ruibal R, Platt-Aloia K 1976 Skin structure and wiping behavior of phyllomedusine frogs. *Copeia* 1976: 283–295.

[cit0008] Boutilier RG, Glass ML, Heisler N 1986 The relative distribution of pulmocutaneous blood flow in *Rana catesbeiana*: effects of pulmonary or cutaneous hypoxia. *The Journal of Experimental Biology* 126: 33–39. 3492587 10.1242/jeb.126.1.33

[cit0009] Brunetti AE, Hermida GN, Luna MC, Barsotti AMG, Jared C, Antoniazzi MM, Rivera-Correa M, Berneck BVM, Faivovich J 2015 Diversity and evolution of sexually dimorphic mental and lateral glands in Cophomantini treefrogs (Anura: Hylidae: Hylinae). *Biological Journal of the Linnean Society* 114: 12–34.

[cit0010] Buttemer WA 1990 Effect of temperature on evaporative water loss of the Australian tree frogs *Litoria caerulea* and *Litoria chloris*. *Physiological Zoology* 63: 1043–1057.

[cit0011] Centeno FC, Antoniazzi MM, Andrade DV, Kodama RT, Sciani JM, Pimenta DC, Jared C 2015 Anuran skin and basking behavior: The case of the treefrog *Bokermannohyla alvarengai* (Bokermann, 1956). *Journal of Morphology* 276: 1172–1182. 26129989 10.1002/jmor.20407

[cit0012] Christian K, Parry D 1997 Reduced rates of water loss and chemical properties of skin secretions of the frogs *Litoria caerulea* and *Cyclorana australis*. *Australian Journal of Zoology* 45: 13–20.

[cit0013] De Lisle SP, Rowe L 2015 Independent evolution of the sexes promotes amphibian diversification. *Proceedings of the Royal Society of London B: Biological Sciences* 282: 20142213. 10.1098/rspb.2014.2213 PMC4345436 25694616

[cit0014] Delfino G, Alvarez BB, Brizzi R, Cespedez JA 1998 Serous cutaneous glands of Argentine *Phyllomedusa* Wagler 1830 (Anura: Hylidae): secretory polymorphism and adaptive plasticity. *Tropical Zoology* 11: 333–351.

[cit0015] Delfino G, Brizzi R, Nosi D, Terreni A 2002 Serous cutaneous glands in New World hylid frogs: an ultrastructural study on skin poisons confirms phylogenetic relationships between *Osteopilus septentrionalis* and *Phrynohyas venulosa*. *Journal of Morphology* 253: 176–186. 12112131 10.1002/jmor.1119

[cit0016] Delfino G, Drewes RC, Magherini S, Malentacchi C, Nosi D, Terreni A 2006 Serous cutaneous glands of the Pacific tree-frog *Hyla regilla* (Anura, Hylidae): patterns of secretory release induced by nor-epinephrine. *Tissue & Cell* 38: 65–77. 16423375 10.1016/j.tice.2005.11.002

[cit0017] Duellman WE, Trueb L 1986 *Biology of amphibians*. Baltimore, MD: Johns Hopkins University Press.

[cit0018] Fox H 1986a Dermis. In: Bereiter-Hahn J, Matoltsy AG, Sylvia R, eds. *Biology of the integument*. Berlin: Springer, 111–115.

[cit0019] Fox H 1986b Epidermis. In: Bereiter-Hahn J, Matoltsy AG, Sylvia R, eds. *Biology of the integument*. Berlin: Springer, 78–110.

[cit0020] Geidies H 1954 Abgeanderte Azan-Methoden. *Mikrokosmos* 42: 239–240.

[cit0021] Geise W, Linsenmair KE 1986 Adaptations of the reed frog *Hyperolius viridiflavus* (Amphibia, Anura, Hyperoliidae) to its arid environment: II. Some aspects of the water economy of *Hyperolius viridiflavus nitidulus* under wet and dry season conditions. *Oecologia* 68: 542–548. 28311710 10.1007/BF00378769

[cit0022] Goldner J 1938. A modification of the Masson trichrome technique for routine laboratory purposes. *The American Journal of Pathology* 14: 237–243. PMC1964940 19970387

[cit0023] Greven H, Zanger K, Schwinger G 1995 Mechanical properties of the skin of *Xenopus laevis* (Anura, Amphibia). *Journal of Morphology* 224: 15–22. 7723046 10.1002/jmor.1052240103

[cit0024] Heatwole H 1963 Ecologic segregation of two species of tropical frogs in the genus *Eleutherodactylus*. *Caribbean Journal of Science* 3: 17–23.

[cit0025] Katz U 1986 The role of amphibian epidermis in osmoregulation and its adaptive response to changing environment. In: Bereiter-Hahn J, Matoltsy AG, Sylvia R, eds. Biology of the integument Berlin: Springer, 472–498.

[cit0026] Kobelt F, Linsenmair KE 1986 Adaptations of the reed frog *Hyperolius viridiflavus* (Amphibia, Anura, Hyperoliidae) to its arid environment: I. The skin of *Hyperolius viridiflavus nitidulus* in wet and dry season conditions. Oecologia 68: 533–541. 10.1007/BF00378768 28311709

[cit0027] Kun SL 1959 Observations on the seasonal changes in the histological structure of the skin of the giant toad (*Bufo bufo gargarizans* Cantor). *Acta Zoologica Sinica* 11: 313–330.

[cit0028] Le Quang Trong Y 1971 Étude de la peau et des glandes cutan-tées de quelques Amphibiens du genre *Phrynobatrachus*. *Bulletin de l’Institut fond d’Afrique Noire* 33: 987–1025.

[cit0029] Le Quang Trong Y 1975 Étude de la peau et des glandes cutanées de quelques amphibiens du genre *Ptychadena*. *Annales de l’Universite d’Abidjan Serie E, Ecologie* 8: 31–52.

[cit0030] Lili J, Chuan T, Shulan L 2013 Comparative histological observation of skin in male and female frog (*Rana dybowskii*) during breeding season. Chinese Agricultural Science Bulletin 32: 23–26.

[cit0031] McClanahan L Jr, Baldwin R 1969 Rate of water uptake through the integument of the desert toad, *Bufo punctatus*. *Comparative Biochemistry and Physiology* 28: 381–389. 5777385 10.1016/0010-406x(69)91351-6

[cit0032] Navas C, Antoniazzi M, Jared C 2004 A preliminary assessment of anuran physiological and morphological adaptation to the Caatinga, a Brazilian semi-arid environment. In: Morris S, Vosloo A, eds. *Animals and environments: proceedings of the third international conference of comparative physiology and biochemistry*. Cambridge and Oxford: Elsevier, 298–305.

[cit0033] Ponssa ML, Barrionuevo JS, Pucci Alcaide F, Pucci Alcaide A 2017 Morphometric variations in the skin layers of frogs: an exploration into their relation with ecological parameters in *Leptodactylus* (Anura, Leptodactylidae), with an emphasis on the Eberth-Kastschenko Layer. *Anatomical Record* 300: 1895–1909. 10.1002/ar.23640 28681539

[cit0034] R Core Team. 2018 R: A language and environment for statistical computing. Vienna, Austria: R Foundation for Statistical Computing ISBN 3-900051-07-0. Available at: http://www.R-project.org/

[cit0035] Rosenberg M, Warburg MR 1995 Changes in structure and function of ventral epidermis in *Hyla arborea savignyi* Aud. (Anura; Hylidae) throughout metamorphosis. *Acta Zoologica* 76: 217–227.

[cit0036] Roth JJ 1973 Vascular supply to the ventral pelvic region of anurans as related to water balance. *Journal of Morphology* 140: 443–460. 30352495 10.1002/jmor.1051400405

[cit0037] Schwinger G, Zanger K, Greven H 2001 Structural and mechanical aspects of the skin of *Bufo marinus* (Anura, Amphibia). *Tissue & Cell* 33: 541–547. 11949790 10.1054/tice.2001.0208

[cit0038] Sever DM 1976 Morphology of the mental hedonic gland clusters of plethodontid salamanders (Amphibia, Urodela, Plethodontidae). *Journal of Herpetology* 10: 227–239.

[cit0039] Sever DM 1989 Caudal hedonic glands in salamanders of the *Eurycea bislineata* complex (Amphibia: Plethodontidae). *Herpetologica* 45: 322–329.

[cit0040] Shoemaker VH, Balding D, Ruibal R, McClanahan LL Jr 1972 Uricotelism and low evaporative water loss in a South American frog. *Science* 175: 1018–1020. 5009394 10.1126/science.175.4025.1018

[cit0041] Toledo R, Jared C 1993 Cutaneous adaptations to water balance in amphibians. *Comparative Biochemistry and Physiology Part A: Physiology* 105: 593–608.

[cit0042] Tracy CR, Christian KA, Tracy CR 2010 Not just small, wet, and cold: effects of body size and skin resistance on thermoregulation and arboreality of frogs. *Ecology* 91: 1477–1484. 20503879 10.1890/09-0839.1

[cit0043] Voyles J, Young S, Berger L, Campbell C, Voyles WF, Dinudom A, Cook D, Webb R, Alford RA, Skerratt LF, Speare R 2009 Pathogenesis of chytridiomycosis, a cause of catastrophic amphibian declines. *Science* 326: 582–585. 19900897 10.1126/science.1176765

[cit0044] Wake DB, Vredenburg VT 2008 Are we in the midst of the sixth mass extinction? A view from the world of amphibians. *Proceedings of the National Academy of Sciences* 105: 11466–11473. 10.1073/pnas.0801921105 PMC2556420 18695221

[cit0045] Warburg MR, Rosenberg M, Roberts JR, Heatwole H 2000 Cutaneous glands in the Australian hylid *Litoria caerulea* (Amphibia, Hylidae). *Anatomy and Embryology* 201: 341–348. 10839630 10.1007/s004290050323

[cit0046] Weichert CK 1945 Seasonal variation in the mental gland and reproductive organs of the male *Eurycea bislineata*. *Copeia* 1945: 78–84.

[cit0047] Wenying W, Pipeng L, Yuyan L, Zhengyan Z, Yong W 2011 Comparative histological observation of the skin in male and female frog (*Rana amurensis*) during breeding season. *Chinese Journal of Wildlife* 3: 141–145.

[cit0048] Zanger K, Schwinger G, Greven H 1995 Mechanical properties of the skin of *Rana esculenta* (Anura, Amphibia) with some notes on structures related to them. *Annals of Anatomy* 177: 509–514.

