# Supplementary material for: Examining the relationship between sexual dimorphism in skin anatomy and body size in the white-lipped treefrog, Litoria infrafrenata (Anura: Hylidae)
Source: Zool J Linn Soc. 2019 Nov 6;186:491–500. doi: 10.1093/zoolinnean/zly070 (PMC7797633; doi:10.1093/zoolinnean/zly070)
Supplement: Supplementary file 1 [file ZJLS-186-491-s001.pdf]

Table S1. Summary statistics for regressions of skin thickness measurements vs. SVL for all specimens, females, and males. Shaded boxes represent significant values ($p < 0.05$).

		All			Females			Males		
Region	Layer	Int	Slope	R^2	Int	Slope	R^2	Int	Slope	R^2
Dorsal	All	-93.65	33.61	0.48	70.04	4.88	0.02	77.06	-1.40	0.02
	Epidermis	73.34	4.20	0.00	208.67	-34.36	0.48	68.90	0.32	0.00
	Spongy Dermis	6.96	19.86	0.16	74.67	5.45	0.05	76.04	-1.58	0.04
	Compact Dermis	-104.46	39.51	0.83	16.99	15.81	0.21	5.40	14.41	0.54
Ventral	All	-81.64	31.19	0.72	33.61	11.25	0.16	39.26	6.12	0.39
	Epidermis	-107.98	54.18	0.33	216.97	-32.60	0.36	61.06	2.56	0.01
	Spongy Dermis	-48.26	30.06	0.71	75.36	4.57	0.04	-8.71	19.30	0.87
	Compact Dermis	-57.06	30.81	0.62	69.63	5.61	0.04	63.18	1.56	0.02
Thigh	All	-103.95	34.33	0.83	-43.53	24.23	0.72	62.85	1.37	0.01
	Epidermis	-143.17	59.46	0.57	16.19	20.35	0.12	90.80	-5.66	0.10
	Spongy Dermis	-43.29	29.40	0.55	-22.82	25.94	0.62	78.32	-2.05	0.08
	Compact Dermis	-114.07	41.89	0.90	-25.53	24.41	0.37	2.62	15.22	0.23

Table S2. Comparing total thickness between body regions.

**All**	Dorsal	Ventral	Thigh
Dorsal			
Ventral	0.29		
Thigh	0.6	0.29	
**Females**			
Dorsal			
Ventral	0.08		
Thigh	0.46	0.12	
**Males**			
Dorsal			
Ventral	0.83		
Thigh	0.83	0.28	

Table S3. Average skin thickness of male and female *Litoria infrafrenata* compared using Kruskal-Wallis tests both on the raw data and residuals from regressions of skin thickness vs. SVL with the pathological specimen (MfN 54646) removed.

		Raw data			Residuals		
Region	Layer	Chi-squared	df	p-value	Chi-squared	df	p-value
Dorsal	All	4.5	1	0.03	0	1	1
	Epidermis	0.13	1	0.72	0.13	1	0.72
	Spongy Dermis	0	1	1	0.13	1	0.72
	Compact Dermis	4.5	1	0.03	0	1	1
Ventral	All	4.5	1	0.03	0	1	1
	Epidermis	4.5	1	0.03	0.13	1	0.72
	Spongy Dermis	4.5	1	0.03	0	1	1
	Compact Dermis	4.5	1	0.03	0	1	1
Thigh	All	4.5	1	0.03	0	1	1
	Epidermis	3.13	1	0.08	0.5	1	0.48
	Spongy Dermis	4.5	1	0.03	0.5	1	0.48
	Compact Dermis	4.5	1	0.03	0	1	1
