# Supplementary figures and images for: Examining the relationship between sexual dimorphism in skin anatomy and body size in the white-lipped treefrog, Litoria infrafrenata (Anura: Hylidae)
Source: Zool J Linn Soc. 2019 Nov 6;186:491–500. doi: 10.1093/zoolinnean/zly070 (PMC7797633; doi:10.1093/zoolinnean/zly070)

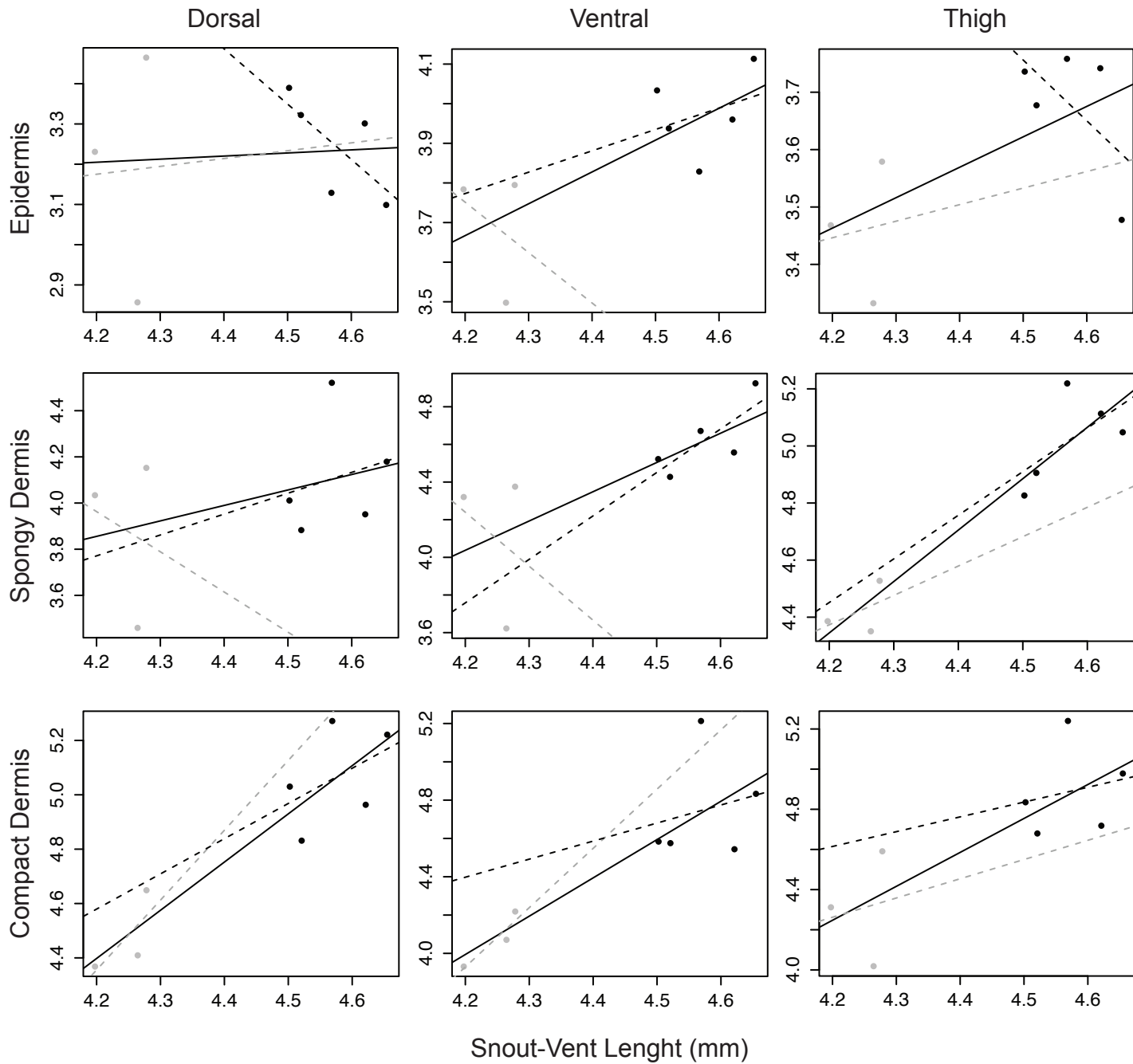

Supplement: Supplementary file 2 [file ZJLS-186-491-s002.pdf]
